# Virtual Empowerment Training: Reaching Grandparent Caregivers During COVID19

**DOI:** 10.1093/geroni/igab046.3040

**Published:** 2021-12-17

**Authors:** Carole Cox

**Affiliations:** Fordham University, Fordham University, New York, United States

## Abstract

COVID-19 has had a devastating impact on minority populations in the United States who have disproportionately been at risk of getting the virus, having severe illness, and dying from it, with these risks most pronounced for older adults. The impact has been particularly severe on the more than on the more than 2, 7 million grandparents raising their grandchildren in the United States. Covid-19 has added profound strains to these families as they struggle with resources and isolation, frequently without assistance as well as the challenge of helping children to navigate online learning. The Virtual Empowerment Training Project, developed as a 7 session pilot program for low income grandparent caregivers in New York includes classes that strengthen parenting, communication, coping with loss and grief, and community empowerment. Participants were given ipads and computer training prior to classes, all conducted through Zoom. Initial data from the 56 participants (M age = 62, Race/ethnicity=Black, 79%, Hispanic, 16%, Income annual 41%<$15,K ) indicate improvement, i.e. a lessening of Negative Affect regarding the grandchild (p < .01) in the quality of their relationships with their grandchildren while also experiencing a decline (p < .01) in their sense of parental efficacy, possibly reflecting their becoming more critical of their own parental skills. Participant evaluations of the course were overwhelmingly positive, with more than 90% finding it extremely helpful, valuable, and eager to share it with others and to become more involved in improving the lives of grandfamilies..

